# A Cross-Sectional Study on Protein Substitutes for Paediatric Phenylketonuria Diet: Time to Pay Attention

**DOI:** 10.3390/nu17111767

**Published:** 2025-05-23

**Authors:** Albina Tummolo, Rosa Carella, Donatella De Giovanni, Vito Di Tullio, Letizia Lorusso, Nicola Bartolomeo

**Affiliations:** 1 Department of Metabolic Diseases and Clinical Genetics, Giovanni XXIII Children Hospital, Azienda Ospedaliero-Universitaria Consorziale, 70126 Bari, Italy; rosa.carella@policlinico.ba.it (R.C.); donatella.degiovanni@policlinico.ba.it (D.D.G.); vditullio11@libero.it (V.D.T.); 2 Interdisciplinary Department of Medicine, School of Medical Statistics and Biometry, University of Bari Aldo Moro, 70121 Bari, Italy; letizia.lorusso@uniba.it; 3 Interdisciplinary Department of Medicine, University of Bari Aldo Moro, 70121 Bari, Italy; nicola.bartolomeo@uniba.it

**Keywords:** micronutrients, PKU, Phenylketonuria, protein substitutes, diet, RDA, requirements, supplementation

## Abstract

**Introduction**: Protein substitutes (PSs) free of phenylalanine (Phe) represent the primary source of proteins and micronutrients in dietary management of classical Phenylketonuria (PKU). Over the last few years, the composition of PSs has undergone rapid and significant improvements, including the development of slow-release amino acid technologies, the introduction of glycomacropeptide-based products, as well as enhancements in taste and the variety of available formulations. However, their micronutrient content has received limited attention. This work aims to analyse the micronutrient composition of all PS formulations available in Italy for paediatric PKU patients and compare their micronutrient contribution to the recommended dietary allowance (RDA) and assess variability among products and age groups. **Materials and Methods**: The content of 28 micronutrients was analysed in 63 PSs, grouped according to the age ranges defined by the RDA guidelines: 0–6 months, 6–12 months, 1–3 years, 3–8 years, 8–14 years. The micronutrient content was standardised for 10 g of protein equivalent (PE). **Results**: Compared to the RDA, many micronutrients were found to be over-supplemented across all age groups, particularly in the 0–6 month group, where peak levels were observed for vitamin K, chromium, and manganese. The 1–3 age group showed the lowest levels of supplementation, with most micronutrients falling within the sub-supplementation range. The variability in supplementation among PSs was broad and showed the highest values in the latter age ranges, reaching maximum levels for biotin and copper. Among different ages, the variability was higher in the first two age ranges, particularly for vitamin A. Choline is not supplemented in many PSs across different age ranges. **Conclusions:** Many micronutrients supplemented in the PSs exceed the RDA for all age groups, with high variability among different PSs and age groups. When prescribing a PS, the daily amount of synthetic proteins of the diet should be considered in order to evaluate the real daily intake of micronutrients in a PKU diet.

## 1. Introduction

Phenylketonuria (PKU, OMIM 261600) is an inborn error of metabolism (IEM) affecting the phenylalanine–tyrosine degradation pathway necessitating the reduction of Phenylalanine (Phe) tissue and plasma levels to prevent long-term neuropsychological sequelae [1]. As for many IEMs, the basic principle of management in PKU is to reduce the unmetabolised substrate concentration by reducing the consumption of nutrients containing such amino acid [2,3]. The dietary interventions required for managing patients with PKU may place them, particularly during growth, at risk for macro- and/or micronutrient deficiency [4]. Special medical formulas that include macro- and micronutrients but omit the offending substrate are used to guarantee protein needs [5,6].

As a percentage ranging from 52% to 80% of daily protein intake is derived from protein substitutes (PSs) [4] a dose higher than the FAO/WHO/UNU [7] is given to compensate for the ineffective absorption of L-amino acids and sub-optimal energy and micronutrient intake [8,9,10]. Many products with different tastes, consistencies, and flavours are available in the market, particularly for paediatric age beyond 1 year, in order to help increase acceptance by patients, attain normal growth, and prevent nutritional deficiencies [11]. Many studies report that PKU children are at risk for micronutrient deficiencies such as vitamin B12, folic acid, selenium, zinc, and iron [12,13,14] because of the low intake of foods rich in these micronutrients. In contrast, other studies report higher than normal plasma levels of some micronutrients under a diet regimen, despite a low intake by natural diet [15,16]. A recent review by our group confirmed conflicting evidence regarding micronutrient status in PKU patients under diet therapy. In particular, some vitamins, like vitamin D, are reported to be lower than normal, whereas folic acid levels were found to be higher than the normal levels in many studies [17], possibly secondary to the high folic acid content of Phe-free L-amino acid supplements [18].

In this context, it has been hypothesised that the above different micronutrient statuses could be not only due to different intakes of food containing the above micronutrients, but also to the different micronutrient content of PSs administered for PKU dietary management. They are, in fact, supplemented with vitamins and minerals, but their composition may vary according to age and formulation. Furthermore, the micronutrient content of PSs is not always considered when prescribing a substitute, nor is the micronutrient status of patients routinely assessed prior to prescription [4,19,20,21]. To our knowledge, no previous studies have systematically evaluated and compared the declared micronutrient composition of all commercially available PS formulations for paediatric PKU patients in a single country using a standardised reference such as the recommended dietary allowances (RDA). Moreover, current regulatory guidelines do not provide clear recommendations regarding micronutrient ranges in PSs, leaving variability in formulation largely unaddressed.

This work aims to analyse the micronutrient content of the PSs available for PKU diet in paediatric age, to compare micronutrient intake provided by each PS with their RDA [22], considering the classic PKU diet regimen, and to analyse their variability among ages and products available.

## 2. Materials and Methods

The micronutrient contents of 63 different products available for paediatric PKU dietary management were analysed and collected in an Excel database. The data regarding the composition of the PS were collected from January to May 2023. To ensure the accuracy and reliability of the information, the most recent product data sheets were reviewed and cross-checked in accordance with the manufacturers’ latest formulations and publicly available databases. We ensured that, since the data-collection period, no significant changes have been made to the formulations of the products. Those introduced into the market after the data-collecting period were not included in the analysis. Among the included PSs, the contents of 28 micronutrients (14 vitamins and 14 minerals) were considered. The micronutrients included in the study and their daily requirements on the basis of age ranges according to RDA [22] are reported in Table S1 (Supplementary Materials).

### 2.1. Protein Substitutes

The micronutrient content was obtained from the product data sheets, officially provided by five companies. All micronutrient values were converted into the unit of measurement used in the RDA references to ensure consistency and comparability. Only products formulated for the paediatric age were included. They were distinguished into five groups, according to the age range in which the RDA of micronutrients is distinguished: 0–6 months, 6–12 months, 1–3 years, 3–8 years, 8–14 years. Table 1 reports the number of products available for age range comprising both traditional L-amino acid formulas, slow-release amino acid formulations and glycomacropeptide-based PS; and their formulation: powder, liquids, micro-tablets, micro-granules, bars, tablets, and puddings.

In the first three age groups, there are only two formulations, powder and liquid, with the first being the most representative. The 3–8 years group includes 55 products, of which 32 are powders, 15 are liquids, and the remaining 8 have different formulations: micro-tablets (2), bars (4), tablets (1), and pudding (1). The 8–14 years group includes 50 products, of which 27 are powders, 15 are liquids, and the remaining formulations are equally represented as in the previous age range.

Some products have been included in more than one group because their indication covers a more extended age range than the first one in which they are included (Table 2). For this reason, the last two groups contain the highest number of products because they also include PSs from the previous groups, whose prescription begins in the younger age categories and for which there is no upper age limit for prescribing. The 0–6 months group includes two products labelled “from birth”, meaning it is possible to prescribe the same product from the newborn period onwards. Similarly, the 6–12 months group includes 2 products labelled “from 6 months”, the 1–3 years group includes two products from one year onwards, and the 3–8 years group includes thirty-one products from three years, 4 from four years and one from 7 years. In the last age group, two products can be prescribed from 8 years, one of which can also be prescribed during pregnancy. Table 2 reports the products by age range and their indications related to the age range.

Table 3 provides a clearer layout of products by age range. Specifically, it distinguishes infant formulas (prescribable from birth), second stage formulas (follow-on formulas prescribed from 6 months), third stage formulas (prescribed from 3 years), and others, referring to formulas that do not fall into the categories above.

### 2.2. Estimation of Micronutrient Daily Requirement

As there are no specific indications of micronutrient requirements for the PKU diet regimen, this value was derived from the total daily protein requirement for the PKU diet, which, according to the European PKU guidelines [4], is 40% higher than the safe daily intake levels defined by the WHO [7] for the general population.

Assuming that, in the classic PKU diet regimen, the PS provided between 52% and 80% of the total protein needs [4,23,24,25], the PS was considered to contribute an average of 66% of the total daily protein intake (Table 4).

The intake of micronutrients from PSs was calculated based on the amount of product required to meet 66% of the total daily protein requirements for each age group. Subsequently, the estimated daily intake of each micronutrient from the PS was expressed as a percentage of the corresponding RDA [22] to compare the intake from the PS and the daily needs. Micronutrient intake and its comparison with RDA were calculated under the assumption of full adherence by patients of each age group to the prescribed protein amounts, as recommended by clinical guidelines.

### 2.3. Micronutrient Content Calculation and Statistical Analysis

In order to standardise comparisons among different formulations, the micronutrient content was calculated for 10 g of protein equivalent (PE) contained in each PS [26].

The normality of the micronutrient distributions was assessed using the Shapiro–Wilk test [27,28]. Since most variables did not follow a normal distribution, data were summarised using the median and interquartile range (IQR). To compare micronutrient levels across different age groups, a non-parametric Kruskal–Wallis (KW) test [29,30] was performed to assess differences in median values among the age classes. The variability of micronutrient values across age groups was analysed using Levene’s test, which evaluates differences in dispersion between groups. Micronutrient content among different PSs was compared using the coefficient of variation calculated in each group for each micronutrient as the ratio between standard deviation and mean per 100 (CV = (σ/μ) × 100). The coefficient of variation (CV) was used to assess the relative variability of micronutrient content among different supplement brands. Although there are no specific guidelines for CV thresholds in the context of food supplements, in statistical practice, a CV below 10% is generally considered indicative of low variability, a CV between 10% and 20% suggests moderate variability, while a CV above 20% indicates high variability [31] and, in our case, poor consistency in nutrient content across different brands. All statistical tests were two-tailed, and a *p*-value < 0.05 was considered statistically significant. Statistical analyses were performed using Jamovi Computer Software (Version 2.6).

## 3. Results

### 3.1. Comparison of Micronutrient Intake by Age Range with RDA

**Figure 1:**
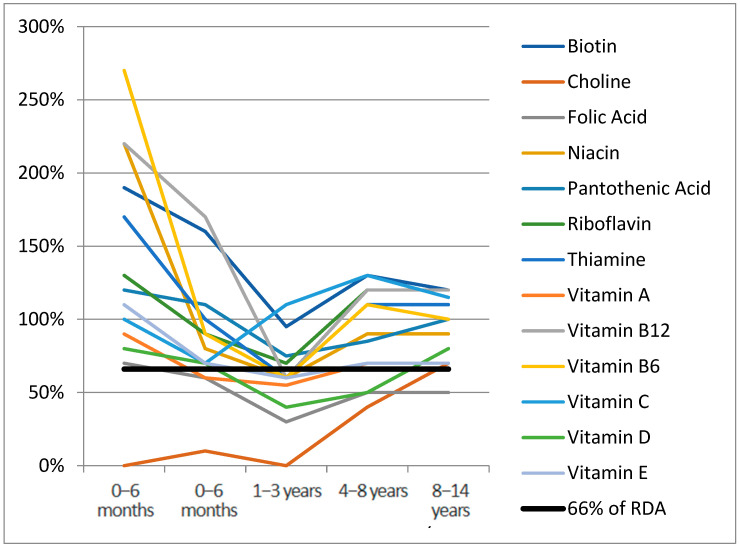
Vitamins median RDA% for age range.

**Figure 2:**
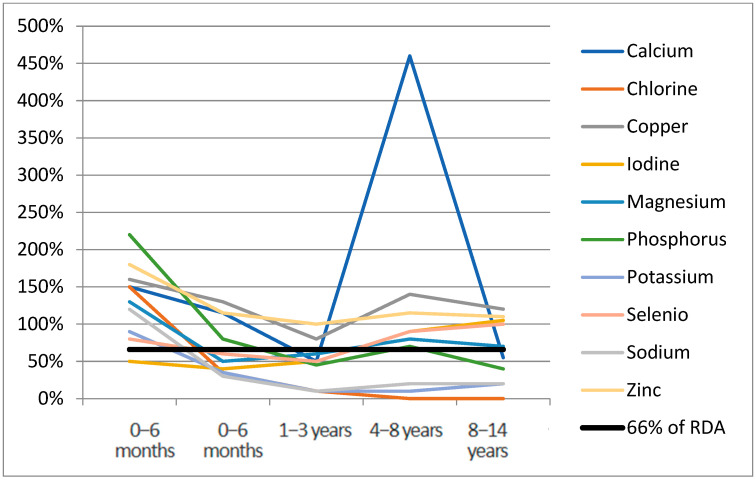
Minerals median RDA% for age range.

Figure 1 and Figure 2 report the median of micronutrient intake expressed as a percentage of the RDA, by age range for vitamins and minerals, respectively. Values of micronutrient intake coverage percentages compared with RDA and expressed as median, interquartile range, and range for each micronutrient across groups are reported in Table S2 (Supplementary Materials). Considering the median nutrient requirement coverage percentages specific to age, in the 0–6 months age range, the highest values among vitamins were observed for vitamin K (1060%) (not reported in Figure 1 to improve the graphical display of other distributions), which drops to 30% in the 1–3 years age range. In this first age range, choline’s median coverage percentage was 0%. Among minerals (Figure 2), chromium (5180%), iron (1960%), manganese (8140%), and molybdenum (970%) were the highest supplemented in the age range of 0–6 months (not reported in Figure 2). In this group, chlorine, iodine, phosphorus, potassium, and sodium fell under the 66% daily requirement in at least two age ranges, with potassium and sodium being the lowest supplemented (Table S2). In the 6–12 months group, all the micronutrients exceeded 66% of RDA; only choline was not supplemented. In the 1–3 years group, eight vitamins (57%) and ten minerals (71%) were found under the 66% average coverage. For the range 3–8 years, chlorine was the lowest represented mineral (0%), followed by sodium (20%) and potassium (10%). In the 8–14 years group, in 28% of cases (4/14), the mineral supplementation was lower than 66% of the daily requirement. Among vitamins, only folic acid (50%) and vitamin K (60%) were undersupplemented.

Median RDA% for vitamin K (1060%) not reported for age range 0–6 months.

Median RDA% for chromium (5180%), iron (1960%), manganese (8140%), molybdenum (970%) not reported for age range 0–6 months.

### 3.2. Variability of Micronutrient Content Among Different PSs

The CV calculated for each microelement among products of the same age group is reported in Table 4. The CV was less than or equal to 20% (low-moderate) in 13% of cases (19/140) across all age range groups. The vast majority, 10/19 of low-moderate CV, were observed in the 0–6 months group (biotin 20%, folic acid 17%, riboflavin 19%, vitamin D 10%, calcium 20%, copper 16%, magnesium 20%, phosphorus 14%, potassium 19%, selenium 18%), 3/19 in the 6–12 months group (folic acid 14%, magnesium 16% and phosphorus 14%), and 6/19 in the 1–3 years group (riboflavin 14%, vitamin B6 15%, calcium 15%, iron 20%, magnesium 16%, phosphorus 17%). No coefficients of variation less than or equal to 20% were observed in the last two age groups, which in fact showed the highest variability (Table 5), with values exceeding 100% for biotin in both the 3–8 years and in the 8–14 years groups (134%, 128% respectively), and for copper in the 8–14 years (595%).

### 3.3. Variability of Single Micronutrient Content Among Different Age Ranges

The analysis of micronutrient content across different age groups revealed significant variations for several micronutrients. The Kruskal–Wallis test indicated statistically significant differences (*p* < 0.001) for pantothenic acid, calcium, chlorine, iron, magnesium, manganese, potassium, vitamin A, vitamin C, vitamin D, and vitamin E across age classes. Notably, iron content decreased from 5.8 mg [4.1–6.3] in 0–6 months to 3.6 mg [3.3–3.8] in 8–14 years, while vitamin A intake declined from 431 mcg [322–500] in 0–6 months to 159 mcg [139–180] in 8–14 years. Regarding the variability of nutrient content across age groups, assessed using Levene’s test [32], significant differences (*p* < 0.05) were found for folic acid, biotin, iodine, vitamin A, vitamin B12, vitamin C, and vitamin E, indicating that the dispersion of values differs significantly between age groups (Table 6).

**Figure 3:**
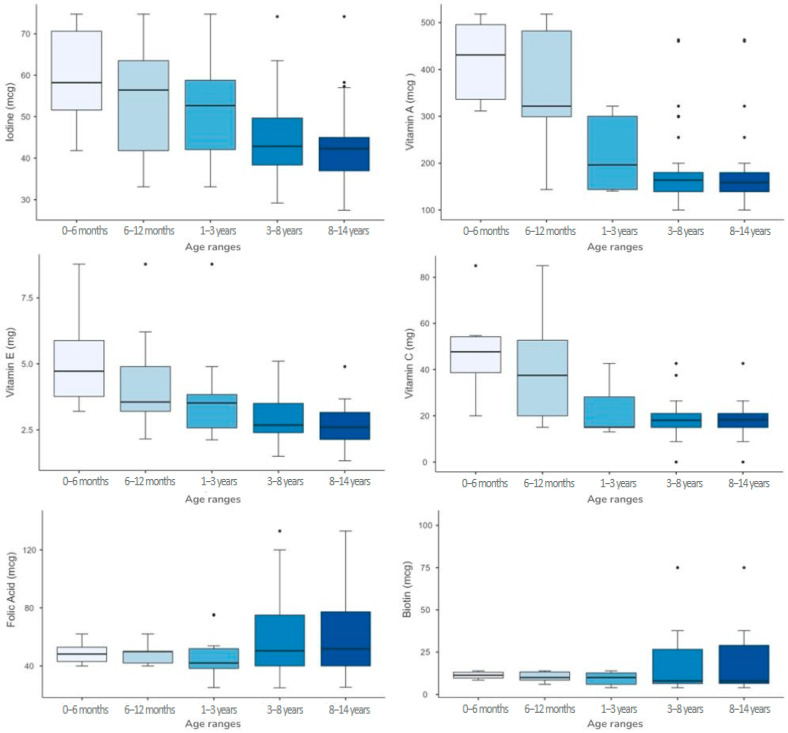
Boxplot representation of micronutrients with non-homogeneous variability across age groups.

For several micronutrients, greater variability was observed in the 0–6 months and 6–12 months age group, compared to older children. For instance, Vitamin A showed a wider IQR in younger age groups (0–6 months: 431 [322–500] mcg; 6–12 months: 322 [260–487] mcg) than in older children (8–14 years: 159 [139–180] mcg). A similar trend was found for Vitamin C (0–6 months: 47.7 [37.3–54.7] mg vs. 8–14 years: 18.3 [15–21] mg) and Vitamin E (0–6 months: 4.7 [3.5–6.2] mg vs. 8–14 years: 2.6 [2.1–3.2] mg), suggesting that formulations for infants tend to be more heterogeneous across different brands (Figure 3). Conversely, greater variability was observed in the older age groups for some micronutrients. For example, folic acid had a relatively narrow IQR in the 0–6 months group (48.2 [42–53.8] mcg) and 6–12 months group (49.9 [42–51]) but was more variable in the 3–8 years and 8–14 years groups (50.4 [40–75.2] and 51.8 [40–78.5] mcg, respectively). Similarly, biotin showed higher heterogeneity in 8–14 years (8 [6.4–30.1] mcg) compared to 0–6 months (11.4 [9.6–13.3] mcg). These findings indicate that, while infant formulations show greater brand-to-brand inconsistencies for some micronutrients, older children’s formulations may also exhibit high variability for others (Figure 3).

## 4. Discussion

Alongside the growing expertise and multidisciplinary approach in managing PKU diet, manufacturers of PSs greatly improved the quality and the composition of amino acid formulas [33]. This evolution has also improved patient compliance and, consequently, disease management and outcomes. At the same time, the availability of many products has introduced the necessity of choosing the best formula based on the patient’s characteristics in order to have the most tailored nutritional approach.

This study sheds light on the content of micronutrient in all the available PSs for paediatric PKU diet age in Italy. To the best of our knowledge, it is the first time that the daily micronutrient content has been analysed, considering the amount provided by the PS in a classic PKU diet regimen. Many micronutrients resulted as oversupplemented in relation to RDA in all age groups, particularly in the 0–6 months group with a peak for vitamin K, chromium, manganese, molybdenum, and iron.

The 1–3 age group was the least supplemented compared to other groups, with most of the micronutrients resulting in sub-supplementation, particularly minerals. The variability in the supplementation of each micronutrient among different age groups and formulas is high and reaches maximum values for biotin and copper in the 4–8 years and 8–14 years groups.

Several formulas are completely lacking in specific micronutrients, particularly those indicated for the late infancy and adolescence age ranges. Among these, choline, found to be absent or under-supplemented in almost all of the age groups and formulas evaluated, plays a key role in several physiological processes, including brain development and neurotransmitter synthesis. It is a precursor of phospholipids, which are essential components of cell membranes and myelin sheaths in the central nervous system [34,35]. Since natural dietary sources of choline are limited or absent in the PKU diet, supplementation through PSs is essential to ensure adequate intake of this nutrient.

On the other hand, for some micronutrients, such as sodium, chloride, potassium, vitamin C, and vitamin K, supplementation may not be essential, as sodium, potassium, chlorine, and vitamin C are primarily obtained through the allowed foods (fruits and vegetables) in a PKU diet and thus, in most cases, a low PS content should not pose at risk of undersupplementation. However, in cases of very low Phe tolerance, even fruits and vegetables may be restricted, thus limiting the contribution in their intake from the natural food.

With regard to vitamin K, after the first year of life, when the gut microbiota has reached full colonisation, supplementation is not necessary as endogenous production is sufficient to meet the body’s requirements [36]. However, studies on isotopic tracing techniques on vitamin K report a high turnover rate of this vitamin, with up to 70% of the oral dose being excreted in the bile and urine in a few days [37]. In an Italian study on trace elements in starter infant formulas, chromium, a nonessential element, was found at a safe low plasma level, whereas iron and manganese were many times higher than the RDA. Authors advocate increasing awareness of the potential risks of excessive manganese and iron supplementation in infant-support formulas [38].

Indeed, the comparison of the above micronutrient intake provided through a PKU formula, within a PKU diet regimen and a standard infant formula in a free diet regimen, highlights very few differences, indicating that the micronutrient oversupplementation in this age range may also involve non-PKU infants [39,40,41].

Evidence regarding micronutrient overassumption and its effects on human health is underreported. Potential adverse outcomes of chronic micronutrient over-supplementation may include a multiorgan involvement causing hepatotoxicity, nephrotoxicity, disruptions in mineral homeostasis, and interference with essential metabolic pathways [42].

In particular, it has been reported that oversupplementation of vitamin A can determine irritability, bulging fontanelle, liver damage, growth retardation, and increased intracranial pressure [43]. Also iron, if taken in excessive quantities, can be dangerous. The most common symptoms include vomiting, diarrhea, abdominal pain and, in the most serious cases, shock and liver damage [40]. Excessive zinc intake can cause nausea, interfere with copper absorption, and weaken the immune system [44].

Among the European Union countries, the PS vitamins and minerals composition for the PKU diet is governed by Commission Delegated Regulation (EU) 2016/128 [45]. A recent study by Verduci et al. [46] analysed the nutritional composition of Phe-free infant PSs, macronutrients and micronutrients, and functional components available for PKU dietary management in Italy. In this study, iron, calcium, and zinc contents vary among formulas, but comply with regulation requirements. Selenium is slightly below the minimum requirement, as well as vitamin D, whose content does not comply with the required range reported in the regulation for three out of the seven infant formulas. All the PSs contain an adequate amount of vitamin B12, thus complying with the Commission Delegated Regulation (EU) 2016/128. In this study, however, the micronutrient content of the formulas prescribed between 0 and 12 months was assessed without considering the micronutrient intake as strictly related to the amount of the synthetic proteins administered daily. In a retrospective study by Evans et al. on 78 subjects aged 1–16 years, the median intake of all of the micronutrients studied was found to be higher than 200% of the reference nutrient intake (RNI), and dietary intake exceeded the upper tolerable intakes for zinc, copper, and folic acid [47]. For PS indicated for later ages in PKU patients, there are no studies investigating micronutrient content. In a recent study by Arslan et al. [48], the nutritional profiles of PS used in Turkey were analysed to compare their contents with equivalent products in the market. Data on macronutrient content were available. However, no micronutrient information was reported, and they could not be available from PS labels [49]. A systematic review by Ilgaz reported evidence from eight papers regarding micronutrient intake and status after diet discontinuation during BH4 therapy. Significant decreases were reported in vitamin (OH)D3, vitamin B12, folic acid, iron, and calcium intake with a liberalised diet [50]. Similar changes in intakes of these micronutrients were reported by Brantley et al. after the beginning of BH4 treatment and after the diet was liberalised, along with significant decreases in serum iron, folic acid, and vitamin B12 concentrations compared to the baseline [51]. Lower intakes of calcium, iron, and vitamin B12 were also observed by Hennermann et al. [52], but only in patients who could liberalise their diet without PSs, indicating that PSs are the major means of micronutrient intake in the PKU diet [53].

In a review by Lammardo et al. [16], it is reported that micronutrients in Phe-free l-amino acids increased without efficacy evidence and that the ideal micronutrient profile of Phe-free l-amino acids is undefined. High/low doses of Phe-free l-amino acid supplements may affect micronutrient intake. The authors conclude that recommendations are required to monitor micronutrient status as well as the composition of L-amino acid supplements.

A study on 112 PKU patients suggested a possible link between diet compliance and copper status, finding higher mean serum copper levels in well-controlled patients, but the amount of copper supplementation provided by PSs and compliance with PS assumption was not considered for assessing this micronutrient status. Although copper is not generally considered a critical micronutrient in PKU, the variability in its supplementation across PS products may support the need for regular monitoring during follow-up [54].

Our study results, based on a systematic analysis of all the PSs available on the Italian market, represent, to the best of our knowledge, the first attempt to determine the daily intake of all the micronutrients based on the PS content in the context of a classic PKU diet. This study has some limitations. Variations in the daily prescription of PSs depending on Phe tolerance/type of PKU were not considered. We acknowledge that individual variability in the contribution of PSs to total protein intake may influence the adequacy of micronutrient coverage. Furthermore, compliance may vary significantly across different paediatric age groups and represents an important issue that should be considered when interpreting the actual nutritional status and risk of deficiency or excess. Also, the actual content in fruits and vegetables rich in vitamin C and B should be taken into account for the daily micronutrient intake calculation.

While our analysis focuses on micronutrients, we recognise that the amino acid profiles of these products are also essential for ensuring nutritional adequacy and should be addressed in future dedicated studies, alongside a comparison between estimated data and actual patient diet compliance.

Our estimation does not take into account the bioavailability and absorption of microelements provided by PSs, and does not associate the micronutrient intake with their status in the plasma, whose monitoring should be included in the follow-up protocol of PKU patients. In PKU, there is no simple relationship between dietary intake and nutritional status, and there are many independent and interrelated factors other than quantitative nutritional intake that should be considered. Vitamins and minerals in medical foods are not bound to proteins and other macronutrients, and thus absorption and utilisation may be lower than those bound to food sources. Also, the RDA is based on the absorption profile of vitamins and oligo elements bound to macronutrients, and the absorption of those added to formulas may be different. Diets must be increasingly personalised and tailored to the clinical outcome, aiming to optimise the overall well-being of the patient, which now extends beyond merely achieving a satisfactory metabolic control of the disease. In this context, the adequacy of micronutrient supplementation can be even more compromised by the age-inappropriateness of some mixtures, which can be prescribed from birth or the first months of life until adult age.

## 5. Conclusions

PSs constitute the main nutritional source in most PKU patients’ diets. The daily intake of micronutrients should be considered based on both the intake of natural foods and the percentage of synthetic proteins prescribed in the diet. Manufacturers should ensure that PS products meet the specific micronutrient requirements of PKU patients and should clearly declare the rate of bioavailability of each micronutrient. They should also maximise the production of more age-appropriate formulations to optimise growth and neurological development at each age range.

Assessment of nutritional status before the start of dietary therapy and micronutrient content per 10 g of PE should be part of prescribing a PS. Further studies are needed to assess the pharmacokinetic profile of micronutrients in the PS, quantifying their absorption rate and availability for cellular processes. The consequent micronutrient plasma concentration should also be associated with possible clinical consequences related to abnormal micronutrient intake, which may be unrecognised in the conventional clinical assessment of the PKU patient.

## Figures and Tables

**Table 1:** Formulation of PS by age range.

	Age Range				
	0–6 Months	6–12 Months	1–3 Years	3–8 Years	8–14 Years
**Total number of products**	7	10	14	55	50
**Powders**	6	9	13	32	27
**Liquids**	1	1	1	15	15
**Micro-tablets**	0	0	0	2	2
**Bars**	0	0	0	4	4
**Tablets**	0	0	0	1	1
**Puddings**	0	0	0	1	1

**Table 2:** PS by age range prescribability and age range for RDA.

	Age Range				
Prescribability	0–6 Months	6–12 Months	1–3 Years	3–8 Years	8–14 Years
**0–12 months**	4	4			
**0–3 years**	1	1	1		
**from birth**	2	2	2	2	2
**6 months–5 years**		1	1	1	
**6 months–10 years**		1	1	1	1
**from 6 months**		1	1	1	1
**1–6 years**			2	2	
**1–8 years**			2	2	
**1–10 years**			2	2	2
**from 1 years**			2	2	2
**3–6 years**				3	
**from 3 years**				31	31
**from 4 years**				4	4
**7–14 years**				3	3
**from 7 years**				1	1
**8–14 years**					1
**from 8 years**					1
**from 8 years and pregnancy**					1

**Table 3:** Distribution of infant formulas, second stage formulas, third stage formulas, and other formulas by age range.

	Age Range				
	0–6 Months	6–12 Months	1–3 Years	3–8 Years	8–14 Years
Infant formulas (from birth)	7	7	3	2	2
Second stage infant formulas (from 6 months)	0	3	3	3	2
Third stage formulas (from 3 years)	0	0	0	34	31
Other	0	0	8	16	15

**Table 4:** Daily protein requirements in PKU diet.

	Protein Requirement in PKU (g/day) = Protein Requirement UNU/FAO (g/day) × 140%					
Age Range	Boys		Girls		Average of Total Protein Requirements in the 2 Genders	Average of Protein Intake from PSs in the 2 Genders (66%)
**0–6 months**	14.28	16.24	13.16	15.12	14.70	9.70
**6–12 months**	14.28	16.24	13.16	15.12	14.70	9.70
**1–3 years**	16.24	18.34	15.12	17.78	16.87	11.13
**3–8 years**	23.94	36.26	22.68	36.68	29.89	19.73
**8–14 years**	36.26	56.7	36.68	57.4	46.76	30.86

**Table 5:** Coefficient of variation of each microelement among age ranges.

Micronutrients	Age Range				
	0–6 Months	6–12 Months	1–3 Years	3–8 Years	8–14 Years
**Biotin**	20%	31%	40%	134%	128%
**Choline (mg)**	60%	42%	51%	46%	40%
**Folic Acid (mcg)**	17%	14%	33%	53%	52%
**Niacin (mg)**	53%	57%	32%	47%	49%
**Pantothenic Acid (mg)**	29%	37%	25%	43%	45%
**Riboflavin (mg)**	19%	22%	14%	24%	24%
**Thiamine (mg)**	22%	30%	24%	24%	21%
**Vitamin A (mcg)**	22%	41%	37%	42%	42%
**Vitamin B12 (mcg)**	76%	81%	25%	48%	52%
**Vitamin B6 (mg)**	30%	26%	15%	31%	32%
**Vitamin C (mg)**	45%	56%	51%	37%	35%
**Vitamin D (mcg)**	10%	30%	31%	41%	45%
**Vitamin E (mg)**	40%	48%	50%	30%	31%
**Vitamin K (mcg)**	37%	45%	64%	42%	42%
**Calcium (mg)**	20%	21%	15%	39%	41%
**Chlorine (mg)**	21%	34%	28%	69%	79%
**Chromium (mcg)**	37%	32%	38%	42%	44%
**Copper (mg)**	16%	24%	27%	26%	595%
**Iodine (mcg)**	22%	29%	28%	21%	22%
**Iron (mg)**	22%	27%	20%	29%	31%
**Magnesium (mg)**	20%	16%	16%	34%	34%
**Manganese (mg)**	70%	53%	59%	54%	56%
**Molybdenum (mcg)**	36%	40%	29%	33%	35%
**Phosphorus (mg)**	14%	14%	17%	38%	37%
**Potassium (mg)**	19%	31%	43%	49%	50%
**Selenio (mcg)**	18%	23%	31%	23%	22%
**Sodium (mg)**	28%	33%	53%	75%	83%
**Zinc (mg)**	22%	25%	24%	23%	23%

**Table 6:** Nutrient content across age groups and statistical assessment of differences in central tendency and variability.

Micronutrient	Age Range					Group Differences (*)	Homogeneity of Variability Across Formulations (^)
	0–6 Months	6–12 Months	1–3 Years	4–8 Years	8–14 Years		
**Biotin (mcg)**	11.4 [9.6–13.3]	10 [7.9–13.5]	10 [6–13]	8 [6.4–26.8]	8 [6.4–30.1]	0.788	0.013
**Choline (mg)**	70 [37–97.2]	66.9 [56.7–79.1]	66.9 [36.5–76.8]	100 [72.7–105]	100 [90–113.1]	0.232	0.872
**Folic Acid (mcg)**	48.2 [42–53.8]	49.9 [42–51]	42 [38.2–52.8]	50.4 [40–75.2]	51.8 [40–78.5]	0.483	0.003
**Niacin (mg)**	5 [2.3–7.7]	3.4 [2.2–6.1]	3.4 [2.5–4.1]	4.1 [3.3–5.1]	4 [3.3–5.4]	0.662	0.180
**Pantothenic Acid (mg)**	2.3 [2–3]	2.1 [1.6–2.6]	1.7 [1.3–2]	1.3 [1.2–1.7]	1.3 [1.1–1.5]	<0.001	0.771
**Riboflavin (mg)**	0.5 [0.4–0.5]	0.4 [0.3–0.5]	0.4 [0.3–0.4]	0.4 [0.3–0.4]	0.4 [0.3–0.4]	0.263	0.813
**Thiamine (mg)**	0.4 [0.3–0.4]	0.3 [0.3–0.4]	0.3 [0.3–0.4]	0.3 [0.3–0.4]	0.3 [0.3–0.4]	0.747	0.425
**Vitamin A (mcg)**	431 [322–500]	322 [260–487]	196 [144–300]	164 [139–180]	159 [139–180]	<0.001	0.013
**Vitamin B12 (mcg)**	1 [0.9–1.2]	0.9 [0.7–1.1]	0.8 [0.5–0.9]	0.8 [0.7–0.9]	0.8 [0.6–0.8]	0.035	0.018
**Vitamin B6 (mg)**	0.4 [0.3–0.4]	0.3 [0.3–0.4]	0.4 [0.3–0.4]	0.3 [0.3–0.4]	0.3 [0.3–0.4]	0.325	0.618
**Vitamin C (mg)**	47.7 [37.3–54.7]	37.5 [18.8–53.2]	15.1 [15–32.7]	18 [15–21]	18.3 [15–21]	<0.001	<0.001
**Vitamin D (mcg)**	8.4 [8–8.5]	8 [5.7–8.5]	6.6 [4.2–7.4]	4.2 [3.9–5.3]	4 [2.5–5]	<0.001	0.382
**Vitamin E (mg)**	4.7 [3.5–6.2]	3.6 [3–5.2]	3.5 [2.4–3.9]	2.7 [2.4–3.5]	2.6 [2.1–3.2]	<0.001	<0.001
**Vitamin K (mcg)**	24.9 [21.8–28.5]	21.8 [9.8–28.5]	9.8 [8.7–24.3]	13 [12.2–18.4]	13 [12.1–17.9]	0.100	0.136
**Calcium (mg)**	349 [300–412]	313 [275–392]	313 [285–369]	262 [200–312]	235 [199–280]	<0.001	0.588
**Chlorine (mg)**	276 [255–295]	255 [155–284]	160 [148–200]	110 [14–162]	108 [11.3–160]	<0.001	0.178
**Chromium (mcg)**	13.4 [10.1–16.5]	16 [10.4–17]	10.7 [10–16.5]	10 [8.6–16.7]	10 [7.4–16.8]	0.259	0.793
**Copper (mg)**	0.3 [0.3–0.4]	0.3 [0.3–0.3]	0.3 [0.2–0.4]	0.4 [0.3–0.4]	0.4 [0.3–0.4]	0.668	0.133
**Iodine (mcg)**	58.2 [50–74.1]	56.4 [39.6–66.2]	52.7 [38.3–61.2]	42.9 [38.2–49.8]	42.3 [36.3–45]	0.007	0.028
**Iron (mg)**	5.8 [4.1–6.3]	5.5 [3.9–6.2]	4.5 [4–4.8]	3.6 [3.4–4.2]	3.6 [3.3–3.8]	<0.001	0.336
**Magnesium (mg)**	42.3 [31.5–47.3]	40 [37.9–45.2]	44 [40.1–51.9]	54 [48.4–62.8]	54.8 [49.1–62.8]	<0.001	0.116
**Manganese (mg)**	0.3 [0.1–0.4]	0.4 [0.2–0.4]	0.4 [0.3–0.6]	0.5 [0.3–0.8]	0.5 [0.3–0.8]	0.006	0.125
**Molybdenum (mcg)**	20.5 [11.2–23.6]	12 [10.7–21.6]	12 [10.3–15.9]	14.2 [11.3–17]	12.9 [11.3–17]	0.637	0.266
**Phosphorus (mg)**	229 [225–242]	229 [207–232]	210 [179–230]	183 [138–213]	178 [139–211]	0.008	0.071
**Potassium (mg)**	418 [353–455]	353 [253–451]	213 [195–300]	175 [122–226]	166 [120–225]	<0.001	0.814
**Selenio (mcg)**	12.8 [11.8–14.7]	11.8 [9.6–13.8]	11 [8.6–13.1]	13.4 [11.9–14.8]	13.9 [12.9–14.8]	0.142	0.927
**Sodium (mg)**	139 [133–146]	133 [101–142]	99.4 [90.9–133]	104 [76.4–160]	101 [52.2–145]	0.297	0.108
**Zinc (mg)**	4.1 [3.3–4.5]	3.8 [2.6–4.4]	3.3 [2.6–3.7]	3.3 [2.4–3.6]	3.2 [2.3–3.6]	0.058	0.832

Data shown as median [IQR]. (*) *p*-value (Kruskal–Wallis)—group differences. (^) *p*-value (Levene’s Test)—stability of formulations across age groups.

## Data Availability

Data are contained within the article and Supplementary Materials.

## Supplementary Materials

The following supporting information can be downloaded at: https://www.mdpi.com/article/10.3390/nu17111767/s1. Table S1. Daily requirements of the micronutrients according to age ranges; Table S2. Comparison of micronutrients intake by age range with Recommended Daily Allowance (RDA) as percentage of requirements (central tendency and variability); Table S3. Minimum and maximum content of micronutrients excluding PS in which the micronutrient is absent (indicated in the column “n. of PS with content 0” for each group).

## Abbreviations

The following abbreviations are used in this manuscript:

PS	Protein substitute
PHE	Phenylalanine
PKU	Phenylketonuria
RDA	Recommended dietary allowance
WHO	World Health Organization
IEM	Inborn error of metabolism
RNI	Reference nutrient intakes
