# Supplementary material for: A Cross-Sectional Study on Protein Substitutes for Paediatric Phenylketonuria Diet: Time to Pay Attention
Source: Nutrients. 2025 May 23;17(11):1767. doi: 10.3390/nu17111767 (PMC12157769; doi:10.3390/nu17111767)
Supplement: Supplementary file 1 [file nutrients-17-01767-s001.zip › nutrients-3622681-supplementary.pdf]

Table S1. Daily requirements of the micronutrients according to age ranges.

		Age range				
		0-6 months	6-12 months	1-3 years	4-8 years	8- 14 years
RDA	Biotin (mcg)	5	6	8	12	20
	Choline (mg)	125	150	200	250	375
	Folic Acid (mcg)	65	80	150	200	300
	Niacin (mg)	2	4	6	8	12
	Pantothenic Acid (mg)	1,7	1,8	2	3	4
	Riboflavin (mg)	0,3	0,4	0,5	0,6	0,9
	Thiamine (mg)	0,2	0,3	0,5	0,6	0,9
	Vitamin A (mcg)	400	500	300	400	600
	Vitamin B12 (mcg)	0,4	0,5	0,9	1,2	1,8
	Vitamin B6 (mg)	0,1	0,3	0,5	0,6	1
	Vitamin C (mg)	40	50	15	25	45
	Vitamin D (mcg)	10	10	15	15	15
	Vitamin E (mg)	4	5	6	7	11
	Vitamin K (mcg)	2	3	30	55	60
	Calcium (mg)	200	260	700	100	1.300
	Chlorine (mg)	180	570	1.500	1.900	2.300
	Chromium (mcg)	0,2	5,5	11	15	25
	Copper (mg)	0,2	0,22	0,34	0,44	0,7
	Iodine (mcg)	110	130	90	90	120
	Iron (mg)	0,27	11	7	10	8
	Magnesium (mg)	30	75	80	130	240
	Manganese (mg)	0,002	0,6	1,2	1,5	1,9
	Molybdenum (mcg)	2	3	17	22	34
	Phosphorus (mg)	100	275	460	500	1.250

**Potassium (mg)**	400	860	2.000	2.300	2.500
**Selenio (mcg)**	15	20	20	30	40
**Sodium (mg)**	110	370	800	1.000	1.200
**Zinc (mg)**	2	3	3	5	8

Table S2. Comparison of micronutrients intake by age range with Recommended Daily Allowance (RDA) as percentage of requirements (central tendency and variability)

	Age range									
	**0-6 months**		**6-12 months**		**1-3 years**		**3-8 years**		**8- 14 years**	
	**MEDIAN [Q1-Q3]**	**MIN-MAX**	**MEDIAN [Q1-Q3]**	**MIN-MAX**	**MEDIANA [Q1-Q3]**	**MIN-MAX**	**MEDIAN [Q1-Q3]**	**MIN-MAX**	**MEDIAN [Q1-Q3]**	**MIN-MAX**
**Biotin**	190 [175 - 255]%	0 - 270%	160 [110 - 210]%	0 - 220,%	95 [62,5 - 162,5]%	0 - 190%	130 [90 - 275,]%	0 - 1970%	120 [80 - 410,]%	0 - 1850%
**Choline (mg)**	0 [0 - 35]%	0 - 80%	10 [0 - 40]%	0 - 70%	0 [0 - 17,5]%	0 - 60%	40 [0 - 80]%	0 - 160%	70 [0 - 80]%	0 - 170%
**Folic Acid (mcg)**	70 [60 - 75]%	0 - 90%	60 [50 - 60]%	0 - 80%	30 [22,5 - 40]%	0 - 60%	50 [40 - 70]%	0 - 160%	50 [40 - 70]%	0 - 160%
**Niacin (mg)**	220, [100 - 315]%	0 - 400%	80 [45 - 125]%	0 - 200%	60 [30 - 70]%	0 - 100%	90 [40 - 120]%	0 - 200%	90 [42,5 - 127,5]%	0 - 210%
**Pantothenic Acid (mg)**	120 [105 - 155]%	0 - 210%	110, [67,5 - 127,5]%	0 - 200%	80 [55 - 110,]%	0 - 120%	85 [60 - 110,]%	0 - 260%	100 [80 - 110,]%	0 - 310%
**Riboflavin (mg)**	130 [120 - 150]%	0 - 160%	90 [72,5 - 107,5]%	0 - 120%	80 [60 - 90]%	0 - 100%	120 [90 - 130]%	0 - 220,%	120 [92,5 - 140]%	0 - 230,%
**Thiamine (mg)**	170 [130 - 190]%	0 - 220,%	100 [80 - 127,5]%	0 - 150%	65 [50 - 80]%	0 - 90%	110, [77,5 - 120]%	0 - 200%	110, [72,5 - 120]%	0 - 170%
**Vitamin A (mcg)**	90 [80 - 120]%	0 - 130%	60 [37,5 - 85]%	0 - 100%	55 [50 - 110,]%	0 - 120%	70 [60 - 90]%	0 - 230,%	70 [70 - 90]%	0 - 240%
**Vitamin B12 (mcg)**	220, [205 - 260]%	0 - 850%	170 [107,5 - 187,5]%	0 - 680%	75 [60 - 107,5]%	0 - 120%	120 [80 - 140]%	0 - 390%	120 [90 - 140]%	0 - 410,%
**Vitamin B6 (mg)**	270 [225 - 360]%	0 - 440,%	90 [90 - 117,5]%	0 - 150%	60 [60 - 80]%	0 - 100%	110, [90 - 140]%	0 - 260%	100 [82,5 - 130]%	0 - 250%
**Vitamin C (mg)**	100 [70 - 130]%	0 - 210%	70 [32,5 - 95]%	0 - 160%	110 [102,5 - 137,5]%	0 - 320%	130 [77,5 - 150]%	0 - 340%	115 [62,5 - 130]%	0 - 290%
**Vitamin D (mcg)**	80 [70 - 80]%	0 - 80%	70 [37,5 - 80]%	0 - 80%	40 [30 - 50]%	0 - 60%	50 [30 - 70]%	0 - 130%	80 [42,5 - 97,5]%	0 - 200%
**Vitamin E (mg)**	110, [80 - 135]%	0 - 210%	70 [45 - 90]%	0 - 170%	60 [40 - 70]%	0 - 160%	70 [40 - 100]%	0 - 140%	70 [40 - 80]%	0 - 140%
**Vitamin K (mcg)**	1060 [700 - 1370]%	0 - 1460%	850 [380 - 1097,5]%	0 - 1160%	30 [22,5 - 47,5]%	0 - 110,%	45 [30 - 60]%	0 - 120%	60 [40 - 90]%	0 - 170%
**Calcium (mg)**	150 [145 - 195]%	0 - 220,%	115 [100 - 135]%	0 - 170%	50 [40 - 50]%	0 - 60%	460, [382,5 - 567,5]%	0 - 1050%	55 [40 - 70]%	0 - 130%
**Chlorine (mg)**	150 [115 - 155]%	0 - 190%	35 [22,5 - 50]%	0 - 60%	10 [0 - 10]%	0 - 20%	0 [0 - 12,5]%	0 - 30%	0 [0 - 20]%	0 - 30%
**Chromium (mcg)**	5180 [4885 - 7890]%	0 - 11460%	235 [182,5 - 297,5]%	0 - 420%	105 [55 - 150]%	0 - 220,%	130 [70 - 192,5]%	0 - 280%	110, [70 - 180]%	0 - 250%
**Copper (mg)**	160 [130 - 165]%	0 - 170%	130 [87,5 - 140]%	0 - 160%	95 [60 - 110,]%	0 - 130%	140 [70 - 170]%	0 - 200%	120 [70 - 160]%	0 - 90060%
**Iodine (mcg)**	50 [40 - 60]%	0 - 70%	40 [22,5 - 47,5]%	0 - 60%	60 [40 - 70]%	0 - 90%	90 [67,5 - 102,5]%	0 - 160%	105 [80 - 120]%	0 - 190%
**Iron (mg)**	1960, [1450 - 2210]%	0 - 2510%	45 [32,5 - 50]%	0 - 60%	65 [50 - 70]%	0 - 100%	70 [50 - 80]%	0 - 150%	140 [100 - 140]%	0 - 290%
**Magnesium (mg)**	130 [100 -	0 - 160%	50 [42,5 -	0 - 60%	60 [45 - 67,5]%	0 - 80%	80 [60 -	0 - 140%	70 [50 -	0 - 120%

	145]%		60]%				100]%		80]%	
**Manganese (mg)**	8140, [1770 - 16615]%	0 - 17150%	55 [15 - 60]%	0 - 70%	30 [5 - 47,5]%	0 - 90%	70 [30 - 92,5]%	0 - 160%	70 [30 - 100]%	0 - 190%
**Molybdenum (mcg)**	970, [495 - 1085]%	0 - 1210%	390 [315 - 672,5]%	0 - 810%	75 [60 - 100]%	0 - 140%	110, [90 - 150]%	0 - 260%	110, [90 - 150]%	0 - 260%
**Phosphorus (mg)**	220, [210 - 225]%	0 - 300%	80 [70 - 80]%	0 - 110,%	50 [32,5 - 60]%	0 - 60%	70 [50 - 80]%	0 - 140%	40 [30 - 50]%	0 - 90%
**Potassium (mg)**	90 [80 - 110,]%	0 - 130%	35 [30 - 47,5]%	0 - 60%	10 [10 - 10]%	0 - 20%	10 [0 - 20]%	0 - 30%	20 [10 - 27,5]%	0 - 40%
**Selenio (mcg)**	80 [70 - 90]%	0 - 110,%	60 [42,5 - 67,5]%	0 - 80%	50 [42,5 - 70]%	0 - 110,%	90 [57,5 - 100]%	0 - 130%	100 [60 - 110,]%	0 - 160%
**Sodium (mg)**	120 [115 - 125]%	0 - 210%	30 [22,5 - 40]%	0 - 60%	10 [0 - 12,5]%	0 - 30%	20 [0 - 30]%	0 - 70%	20 [0 - 37,5]%	0 - 100%
**Zinc (mg)**	180 [140 - 215]%	0 - 220,%	115 [90 - 140]%	0 - 150%	110 [75 - 137,5]%	0 - 160%	115 [80 - 140]%	0 - 170%	110, [72,5 - 140]%	0 - 160%

Table S3. Minimum and maximum content of micronutrients excluding PS in which the micronutrient is absent (indicated in the column "n. of PS with content 0" for each group)

	Age range									
	0-6 months		6-12 months		1-3 years		3-8 years		8- 14 years	
**Micronutrients**	**N. of PS with content 0**	**content MIN and MAX**	**N. of PS with content 0**	**content MIN and MAX**	**N. of PS with content 0**	**content MIN and MAX**	**N. of PS with content 0**	**content MIN and MAX**	**N. of PS with content 0**	**content MIN and MAX**
**Biotin (mcg)**	1	[8,5 - 13,9]	1	[6 - 13,9]	3	[4 - 13,9]	7	[4 - 120]	7	[4 - 120]
**Choline (mg)**	4	[26 - 106,3]	5	[26 - 106,3]	9	[25,1 - 106,3]	20	[14,8 - 203]	18	[14,8 - 203]
**Folic Acid (mcg)**	1	[40 - 62]	1	[40 - 62]	3	[25,1 - 75,3]	7	[24,9 - 160]	7	[25,3 - 160]
**Niacin (mg)**	1	[1,8 - 8,2]	1	[1,7 - 8,2]	3	[1,7 - 5,1]	7	[0,4 - 8]	7	[0,4 - 8]
**Pantothenic Acid (mg)**	1	[1,7 - 3,6]	1	[1,2 - 3,6]	3	[0,9 - 2,1]	7	[0,9 - 4]	7	[0,9 - 4]
**Riboflavin (mg)**	1	[0,3 - 0,5]	1	[0,3 - 0,5]	3	[0,3 - 0,4]	7	[0,3 - 0,7]	7	[0,3 - 0,7]
**Thiamine (mg)**	1	[0,3 - 0,5]	1	[0,2 - 0,5]	3	[0,2 - 0,4]	10	[0,2 - 0,6]	10	[0,2 - 0,5]
**Vitamin A (mcg)**	1	[311,5 - 518,2]	1	[143,9 - 518,2]	3	[140,5 - 321,7]	10	[100 - 463,2]	10	[100 - 463]
**Vitamin B12 (mcg)**	1	[0,8 - 3,5]	1	[0,5 - 3,5]	3	[0,5 - 0,9]	7	[0,4 - 2,4]	7	[0,4 - 2,4]
**Vitamin B6 (mg)**	1	[0,2 - 0,5]	1	[0,2 - 0,5]	3	[0,3 - 0,4]	7	[0,3 - 0,8]	7	[0,3 - 0,8]
**Vitamin C (mg)**	1	[20 - 85]	1	[15,1 - 85]	3	[13,1 - 24,7]	11	[0,02 - 42,7]	11	[0,02 - 42,7]
**Vitamin D (mcg)**	1	[6,4 - 8,5]	1	[3,5 - 8,5]	3	[3,5 - 8,5]	10	[1,1 - 9,7]	10	[1,1 - 9,7]
**Vitamin E (mg)**	1	[3,2 - 8,8]	1	[2,2 - 8,8]	3	[2,1 - 8,8]	10	[1,5 - 5,1]	10	[1,3 - 4,9]
**Vitamin K (mcg)**	1	[7 - 30]	1	[7 - 30]	3	[5 - 28,5]	10	[5 - 32,2]	10	[5 - 32,2]
**Calcium (mg)**	1	[279,7 - 454,5]	1	[259,7 - 454,5]	3	[259,7 - 385]	7	[8 - 531,9]	7	[8 - 531,9]
**Chlorine (mg)**	1	[170 - 345,5]	1	[139,8 - 345,5]	5	[139,8 - 271]	20	[0,01 - 255,2]	17	[0,01 - 255,2]
**Chromium (mcg)**	1	[10 - 23,6]	1	[10 - 23,6]	3	[4 - 21,3]	10	[4 - 21,3]	10	[4 - 20,1]
**Copper (mg)**	1	[0,3 - 0,4]	1	[0,2 - 0,4]	3	[0,2 - 0,4]	10	[0,1 - 0,4]	10	[0,1 - 204,3]
**Iodine (mcg)**	1	[41,8 - 74,7]	1	[33,1 - 74,7]	3	[33,1 - 74,7]	10	[29,2 - 74,1]	10	[27,4 - 74,1]
**Iron (mg)**	1	[4 - 7]	1	[3,4 - 7]	3	[3,4 - 6,2]	11	[0 - 7,5]	11	[0,004 - 7,5]
**Magnesium (mg)**	1	[30 - 48]	1	[30 - 48]	3	[31,5 - 56,9]	7	[6,5 - 92,3]	7	[6,5 - 90]
**Manganese (mg)**	1	[0,02 - 0,4]	1	[0,02 - 0,4]	4	[0,02 - 0,9]	11	[0,005 - 1,2]	11	[0,005 - 1,2]
**Molybdenum (mcg)**	1	[9,2 - 25]	1	[9,2 - 25]	3	[9,2 - 22]	10	[9 - 28,7]	10	[9 - 28,7]
**Phosphorus (mg)**	1	[209,8 - 304,5]	1	[197,8 - 304,5]	3	[133,5 - 252,9]	7	[40 - 348,9]	7	[40 - 348,9]
**Potassium (mg)**	1	[300 - 522,1]	1	[224,9 - 522,1]	2	[8,4 - 385,5]	8	[3,5 - 384]	7	[3,5 - 353,1]
**Selenio (mcg)**	1	[10 - 16,6]	1	[8,4 - 16,6]	3	[7,3 - 19,3]	10	[7,3 - 20,5]	10	[7,3 - 20,5]
**Sodium (mg)**	1	[120 - 236,4]	1	[90,9 - 236,4]	4	[1,2 - 233,8]	14	[1,2 - 380]	11	[1,2 - 380]
**Zinc (mg)**	1	[2,4 - 4,5]	1	[2,4 - 4,5]	3	[1,9 - 4,4]	10	[1,5 - 4,4]	10	[1,5 - 4,2]

Differences in content were low in the 0-6 months and 6-12 months, with a minimum delta for copper (0,1 mg). The greatest differences were observed in the later age ranges, reaching the highest values in the 1-3 years group for minerals, such as potassium (377 mg), and in the 3-8 years group for calcium (524 mg), chlorine (0.01 - 255.2 mg), potassium (3.5 - 384 mg), and sodium (1.2 - 380 mg), in the 8-14 years group for calcium (8 - 531.9 mg), phosphorus (309 mg), potassium (380 mg), and sodium (349 mg).

Table S3 also provides the number of products that are completely lacking for a micronutrient for each group. In the 0-6 months and in the 6-12 months age groups choline was absent in 4/7 and in 5/10 products, respectively. All the other micronutrients are absent in only 1 product. In the 1-3 years group, chlorine was absent in 5/14 products and choline in 9/14 products. These two minerals were absent also in 20/55 products in the 3-8 years age range. In the 8-14 years group, chlorine and choline were the least supplemented; the others are almost overlapping the previous age ranges. Products not supplemented at all are 1/7 in the 0-6 months group, 1/10 in the 6-12 months groups, 3/14 in the 1- 3 years group, 7/55 in the 3- 8 years and 7/50 in the 8-14 years groups.
